# Direct structural evidence supporting a revolving mechanism in DNA packaging motors

**DOI:** 10.1007/s41048-020-00115-w

**Published:** 2020-10-31

**Authors:** Yao-Gen Shu, Xiaolin Cheng

**Affiliations:** 1 Wenzhou Institute, University of Chinese Academy of Sciences, Wenzhou, Zhejiang 325001, China; 2 College of Pharmacy; Biophysics Graduate Program; Translational Data Analytics Institute, The Ohio State University, Columbus, OH 43210, USA

Viruses have evolved two strategies to package genome: either assembling a capsid around their genomes or packaging the genomes into a preformed capsid with packaging motors. Most of the double-stranded (ds) DNA viruses (*e*.*g*., tailed bacteriophages and herpesviruses) and dsRNA viruses (*e*.*g*., ϕ6 and ϕ12 bacteriophages) take the latter strategy. The packaging motors are usually called the “terminase” complex, which, in bacteriophages comprises two proteins, termed large (TerL) and small (TerS) terminases (Guo *et al*. 1987b), while in herpesviruses contains three components — pUL15, pUL28 and pUL33. The unique ϕ29 motor complex lacks a TerS but instead contains a 174-nt prohead RNA (pRNA) (Guo *et al*. 1987a). However, it has long been debated whether the viral genome packaging motors operate via a rotatory or revolving mechanism and what is their oligomeric state in action — a hexamer or a pentamer? And now, published in a recent issue of *Protein & Cell*, Yang *et al*. have hopefully ended these 20-year long fervent debates by determining high-resolution structures of the herpesvirus terminase complex to reveal the six-fold symmetry with a potential revolving mechanism (Yang *et al*. 2020).

Molecular motors are enzymes that convert a chemical scalar (ATP) into a physical vector (unidirectional motion). They are also called nanomachines as they are all at the nanoscale. Molecular motor is a mesoscopic prototype of a non-equilibrium system of “soft matter + ATP”. Unlike linear motor kinesin, which consumes one ATP molecule for each 8 nm step with a hand-over-hand mechanism (Li *et al*. 2018), and reversible rotary motor F_o_F_1_-ATPase, which synthesizes/hydrolyzes one ATP molecule for each 120° step with a binding-change-mechanism (Shu and Lai 2008), the framework of the genome packaging motors remains elusive. Although rotational motion with tight mechanochemical coupling was long thought as a common mechanism for the DNA packaging motors (Hendrix 1998; Serwer 2003; Simpson *et al*. 2000), a revolving framework of DNA translocation has recently been proposed for the ϕ29 dsDNA packaging motor (Guo *et al*. 2019; Schwartz *et al*. 2013; Zhao *et al*. 2016).

The oligomeric state of the DNA packaging motors has also been debated for decades. The debate initiated in bacteriophage ϕ29 where low-resolution cryo-EM reconstructions slightly favored 5-fold symmetry averaged structures (Morais *et al*. 2001, 2008; Simpson *et al*. 2000), whereas a large body of biochemical data supported a hexameric ring structure (Guo *et al*. 1998; Ibarra *et al*. 2000; Schwartz *et al*. 2013; Trottier and Guo 1997). The same mystery carries over to bacteriophage T4 where atomic structures of the motor proteins seemed able to fit into both 5- and 6-fold symmetry averaged low-resolution (>30 Å) cryo-EM densities (Sun*et al*. 2008). All these controversies on the composition, architecture, and packaging mechanism are rooted from the lack of a high-resolution three-dimensional structure of the viral packaging motors.

Recently, Yang *et al*. have determined the first atomic structures of a herpesvirus terminase complex in both apo and ATP mimic-bound states (Yang *et al*. 2020). When the three components of the herpesvirus terminase complex — the ATPase/terminase pUL15 and two regulator/fixer proteins, pUL28 and pUL33 — were co-expressed using a baculovirus-based expression system, the terminase complex assembled predominantly into a hexameric ring *in* *vitro*. Each subunit of the hexameric ring is a heterotrimer formed by the three proteins (pUL15, pUL28 and pUL33) interdigitating with each other. The terminase pUL15 folds into an “L” shaped structure, containing five functional domains: N-lasso (residues 1 to 152), strut (residues 153 to 252), ATPase (residues 253 to 413), regulator (residues 414 to 478) and nuclease (residues 479 to 735). Structural analysis also ambiguously identified R346 as the *trans-*acting arginine finger that extends from an ATPase subunit to its adjacent ATP binding pocket to interact with the γ-phosphate.

Although atomic structures of several full-length viral large terminase subunits, such as T4 gp17 (Sun *et al*. 2008) and Sf6 gp2 (Zhao *et al*. 2013), are available, herpesvirus pUL15 is the only TerL structure determined in a potentially “functional” oligomeric state. Therefore, the pUL15 structure provides an important template for understanding the relative orientation of the ATPase motors with respect to their DNA substrates and how the individual subunits interact with each other in their oligomeric state. We performed simple structural superposition of the three monomeric TerL structures using their ATPase domains as a reference. We focused on the three domains common to most DNA packaging motors, the ATPase domain (corresponding to the N-terminal domain of ϕ29 gp16, referred to here as NTD), the regulator domain (corresponding to the linker domain of ϕ29 gp16) and the nuclease domain (corresponding to the C-terminal domain of ϕ29 gp16, referred to here as CTD). As shown in Fig. 1, despite their distant relation in the viral family, all the three ATPase domains are aligned remarkably well while large variations are observed in the spatial arrangements of individual domains. Both the NTD–CTD separation and the orientation of CTD relative to NTD vary among the three TerLs. In herpesvirus pUL15, NTD and CTD make no direct contact with each other while both T4 gp17 and Sf6 gp2 show extensive interactions between the two domains. Another intriguing observation is that the catalytic site of the nuclease domain in pUL15 opens towards the adjacent subunit instead of DNA, suggesting the nuclease activity is inhibited during translocation (Yang *et al*. 2020). This information is, however, not immediately clear from the monomeric structure of either T4 gp17 or Sf6 gp2, but based on the aligned structures, it is evident that both nuclease sites are inaccessible to DNA, and conformational changes would be required for both T4 gp17 and Sf6 gp2 to expose their nuclease sites for DNA cleavage upon packaging.

**Figure 1:**
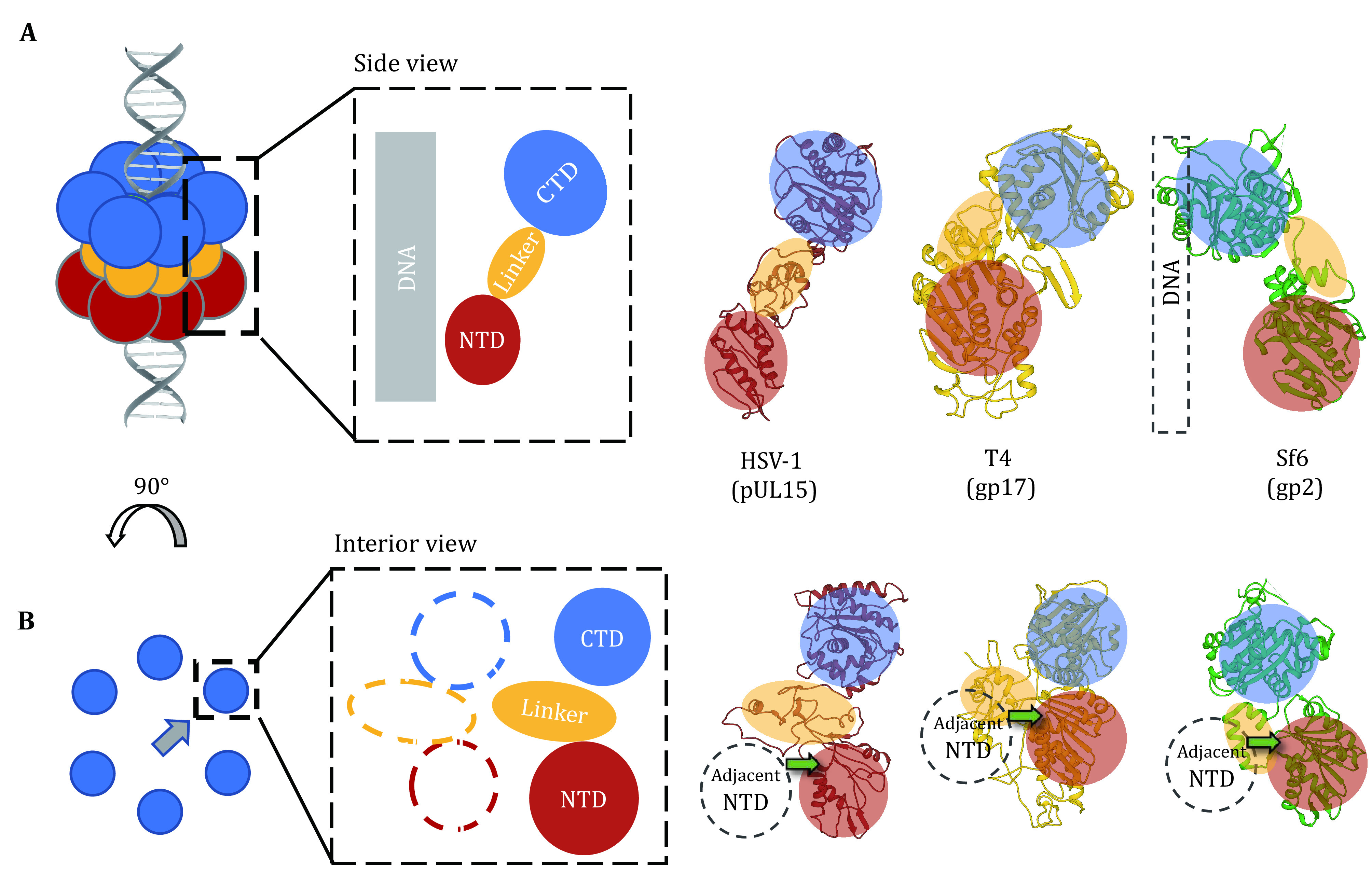
Comparison of viral packaging motor proteins from the side view (**A**) and the interior view (**B**). In the schematic, NTD (N-terminal domain), linker domain and CTD (C-terminal domain) were colored in red, yellow and blue, respectively. The interior view is also denoted by a gray arrow, indicating viewing from the interior of the channel. The green arrows indicate the location of the arginine finger between two neighboring NTDs

Furthermore, these results lend structural support to the revolving mechanism rather than the rotatory mechanism of dsDNA translocation. The six ATPase domains of pUL15 form a central channel with conserved basic patches conducive to DNA binding. A diameter of 39 Å for the central channel, in line with that of the herpesvirus portal (~36 Å in diameter), is much wider than the size of a B-form dsDNA (~20 Å in diameter), which clearly favors the revolving model, since if it were a rotatory motor, a channel smaller than 20 Å in diameter should have been detected because close contact between the channel and the substrate is necessary for a rotational motion to occur. These findings are also supported by molecular dynamics simulations of the ϕ29 connector that revealed a quite heterogeneous distribution of stiff and soft regions of the motor channel, compatible with the one-way revolution model (Kumar and Grubmüller 2014). Interestingly, using building blocks resolved in the hexameric structures, a hypothetical pentameric ring structure could be created to maintain reasonable contact between adjacent subunits, but such a model with much narrower constrictions (19–24 Å) would exclude the revolving translocation mechanism (Yang *et al*. 2020). Taken together, these new structures provide compelling evidence that the hexameric terminase complex uses a revolving mechanism to translocate dsDNA.

Tremendous efforts have been made to determine three-dimensional structures of individual viral proteins and whole herpes virions (Dai and Zhou 2018; Wang *et al*. 2018; Yuan *et al*. 2018), but the resolution was too low to allow meaningful reconstruction of the terminase complex structure. This is partially attributable to the small size of this complex compared to the capsid protein layer as well as the technical difficulties associated with resolving non-icosahedral components in a large icosahedral virus. On the other hand, isolation of an intact terminase complex has been proven extremely challenging. In this study, the authors were able to assemble a hexameric terminase complex *in vitro*, and cryo-EM of the assembled hexamers in combination with a block-reconstruction method (Yuan *et al*. 2018; Zhu *et al*. 2018) has finally led to the first atomic structures of the herpesvirus terminase complex.

Overall, these new atomic structures of the herpesvirus terminase complex provide a reconciling structural view of viral genome packaging in dsDNA viruses. Nevertheless, the detailed revolving mechanism of this dsDNA packaging motor is just beginning to be elucidated because the kinetics of its packaging has not yet been studied quantitatively with a phenomenological model, and many questions related to the kinetic details of the mechanism remain. For example, is the motor a processive motor? Is its mechanochemical coupling tight? Does the revolution include stepping? What is the length of the step? And so on. On the other hand, the entropy of the genome is changing during packaging, so that the reactive force on the packaging motor is no longer constant. Kinetically, the steady state, which is indispensable for quantitative investigation, disappears.

## Conflict of interest

Yao-Gen Shu and Xiaolin Cheng declare that they have no conflict of interest.
